# Supplementary material for: Causal interplay between lactose intolerance and gut microbiota: a combined bidirectional Mendelian randomization and in vivo validation study
Source: Front Nutr. 2026 Jun 1;13:1803337. doi: 10.3389/fnut.2026.1803337 (PMC13265576; doi:10.3389/fnut.2026.1803337)
Supplement: Supplementary file 1 [file Data_Sheet_1.zip › supplementary materials/Reverse/Batch analysis results 20250616224610/forest plot/finngen_R12_E4_LACTONAS.ebi-a-GCST90027788.pdf]

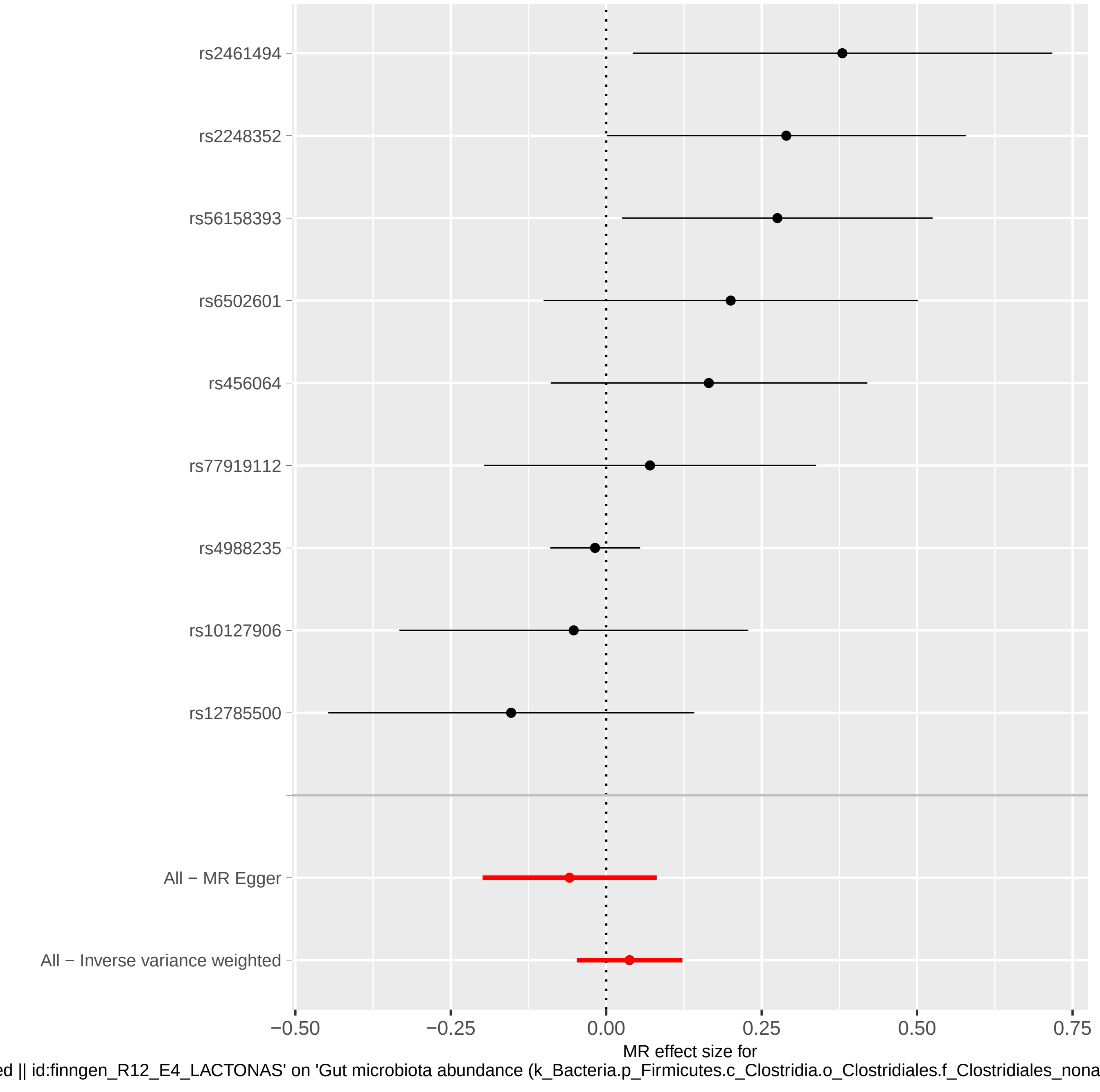

rs2461494
rs2248352
rs56158393
rs6502601
rs456064
rs77919112
rs4988235
rs10127906
rs12785500
All − MR Egger
All − Inverse variance weighted
−0.50
−0.25
0.00
0.25
0.50
0.75
MR effect size for
ed || id:finngen_R12_E4_LACTONAS' on 'Gut microbiota abundance (k_Bacteria.p_Firmicutes.c_Clostridia.o_Clostridiales.f_Clostridiales_nona
